# Mucosal Healing and Clinical Efficacy of Adalimumab in Small Intestinal Crohn’s Disease (SIMCHA Study): Final Results From a Prospective, Open-Label, Single-Arm Study

**DOI:** 10.5152/tjg.2023.22527

**Published:** 2023-06-01

**Authors:** Panu Wetwittayakhlang, Christine Verdon, Michael Starr, Gustavo Drügg Hahn, Petra A. Golovics, Talat Bessissow, Waqqas Afif, Gary Wild, Alain Bitton, Peter L. Lakatos

**Affiliations:** 1 Division of Gastroenterology, McGill University Health Centre, Montreal, Quebec, Canada; 2 Gastroenterology and Hepatology Unit, Division of Internal Medicine, Faculty of Medicine, Prince of Songkla University, Hat Yai, Songkhla, Thailand; 3 Division of Rheumatology, McGill University Health Centre, Montreal, Quebec, Canada; 4 Universidade Federal do Rio Grande do Sul, School of Medicine, Graduate Course Sciences in Gastroenterology and Hepatology, Porto Alegre, Brazil; 5 Department of Gastroenterology, Hungarian Defence Forces, Medical Centre, Budapest, Hungary; 6 Department of Internal Medicine and Oncology, Semmelweis University, Budapest, Hungary

**Keywords:** Crohn’s disease, endoscopic healing, capsule endoscopy, adalimumab, antitumor necrosis factor

## Abstract

**Background:**

Endoscopic healing is a key treatment target in inflammatory bowel disease; few data are available on the clinical and endoscopic efficacy of biological therapy in upper gastrointestinal Crohn’s disease. This study aimed to investigate small bowel mucosal healing and clinical efficacy of adalimumab therapy by video capsule endoscopy in patients with endoscopically active upper gastrointestinal Crohn’s disease.

**Methods:**

This prospective, open-label, single-arm study included Crohn’s disease patients with moderate–severe endoscopic proximal small bowel involvement, defined by a Lewis score >790. Patients were treated with adalimumab monotherapy for 24 weeks. Co-primary outcomes were endoscopic healing, defined as Lewis score <350, and endoscopic response, defined as >50% decrease in Lewis score. Secondary outcomes included clinical (Harvey–Bradshaw index <4) and biomarker remission (fecal calprotectin <250 μg/g, and C-reactive protein <5 mg/L).

**Results:**

A total of 59 Crohn’s disease patients were screened; 17 patients have met eligibility criteria and were enrolled. Endoscopic healing was observed in 8 patients (47.1%) and endoscopic response in additional 5 patients (29.4%) at 24 weeks. Median Lewis score was significantly decreased compared to baseline (1912 vs. 337, *P* = .0005). Eleven of 13 patients (84.6%) with clinical activity achieved clinical remission (baseline: 13/17 vs. week 24: 2/17, *P* < .0001). Nine of 10 patients with elevated C-reactive protein achieved normal C-reactive protein after treatment and the median C-reactive protein significantly decreased from 7.4 to 1.6 mg/L, *P* = .032. In contrast, no change was observed in fecal calprotectin pre- and posttreatment.

**Conclusions:**

Adalimumab induced endoscopic healing and clinical remission in patients with active small bowel Crohn’s disease, with approximately half of the patients achieving endoscopic healing.

**Main Points**

- This study investigated small bowel mucosal healing evaluated by video capsule endoscopy (VCE) and clinical efficacy accessing by Harvey–Bradshaw index and biomarkers [C-reactive protein (CRP) and fecal calprotectin] of adalimumab therapy in patients with moderate–severe endoscopically active upper gastrointestinal Crohn’s disease.
- Approximately half of the patients achieved endoscopic healing (47%) and endoscopic response in an additional one-third of patients (29%). The median Lewis score assessed by VCE was significantly decreased compared to baseline (1912 vs. 337) at 24 weeks of adalimumab therapy.
- 85% of patients with clinical activity achieved clinical remission, and 90% with elevated CRP achieved CRP normalization.

## INTRODUCTION

Crohn’s disease (CD) is a relapsing inflammatory disease that can affect the entire gastrointestinal (GI) tract, however, it primarily affects the terminal ileum and the colon.^1,2^ About 15% of patients with CD have proximal small bowel (SB) involvement.^3^ Although proximal SB lesions are less common, they are associated with a higher risk of complications, stricturing disease, and bowel resection earlier in the course of disease compared with either colonic or distal ileal CD.^4,5^

Endoscopic mucosal healing (MH) is a key treatment target according to current recommendations, a “treat-to-target” strategy has been suggested in inflammatory bowel disease (IBD) to achieve deep disease control, including a combination of clinical and endoscopic healing.^6^ This strategy has been associated with improved long-term outcomes, reduced risks of flare, and surgery in patients with CD.^7,8^ Available evidence is sparse but suggests that SB CD is difficult to treat with a lower probability of MH compared to colonic disease,^9^ even in the current biological era. Only a few studies assessed SB MH using video capsule endoscopy (VCE).^10-12^ A prospective study of 19 CD patients with SB involvement who underwent sequential VCE confirmed that VCE provides reliable information on mucosal changes and also proved the correlation between clinical scores, quality of life, and mucosal inflammation was poor.^11^

Adalimumab, an antitumor necrosis factor (anti-TNF), is reported to be effective in inducing and maintaining MH in CD patients. In the randomized clinical trial, EXTEND study, the rates of colonic MH in patients treated with adalimumab were 27% and 24% at weeks 12 and 52, respectively.^13,14^ Of note, there is little evidence on the efficacy of anti-TNFs on SB MH in CD. Only 1 prospective observational study from Hall et al^15^ demonstrated significant MH in patients treated with anti-TNF or immunomodulator as evaluated by VCE, with approximately 40% achieving MH by week 52.

The assessment of the SB in patients with CD is necessary since it has therapeutic implications and a significant impact on disease outcomes. Video capsule endoscopy allows direct visualization of SB mucosal lesions. Video capsule endoscopy has been reported to be superior to other modalities for diagnosing and evaluating SB involvement in CD,^16,17^ as recommended by American and European guidelines.^1,18^ To date, there are only a handful of studies that reported on the use of VCE in disease monitoring in CD.

In this study, our aim was to report the results of a prospective, open-label, single-arm study on the endoscopic healing (EH) and clinical efficacy of adalimumab treatment in patients with VCE proven- moderate to severe SB CD with active mucosal inflammation.

## MATERIALS AND METHODS

### Study Design and Participants

We conducted a prospective, open-label, single-arm study of adalimumab monotherapy for induction and maintenance treatment in moderate to severe SB CD patients at the McGill University Health Centre, McGill University, between November 2011 and June 2020.

Inclusion criteria were (a) adult patients (>18 years old) diagnosed with CD according to endoscopic, imaging, or histological criteria; (b) Evidence of moderate–severe proximal SB (duodenum, jejunum, or proximal–mid ileum) inflammation on VCE, defined by a total Lewis score (LS) at enrollment >790.

We excluded patients using drugs known to induce SB injury, such as non-steroidal anti-inflammatory drugs, within 3 months before VCE examination. In addition, patients with previous intestinal resection, known intestinal obstruction or current obstructive symptoms, history of SB strictures, previous exposure to biological therapy, and concurrent use of systemic corticosteroids or immunosuppressants (azathioprine, 6-mercaptopurine, methotrexate, and sulfasalazine) were excluded.

### Interventions, Video Capsule Endoscopy

PillCam™ SB2 capsule (Given® Diagnostic Imaging System, Israel) was performed at baseline and 24 weeks after adalimumab treatment. Each patient underwent a patency capsule. Thereafter, a capsule study was performed following 12 hours overnight fast, without using bowel preparation or prokinetics. A sensor belt and recorder were attached to the patient for 8 hours. The recorded digital information was downloaded, and the images were analyzed using RAPID® software. All VCE examinations were evaluated by a single, blinded gastroenterologist with VCE expertise to avoid inter-observer variability and bias.

The LS was used for the assessment of the endoscopic severity of SB CD. The LS was calculated by a specific formula based on 3 endoscopic variables: villous edema, ulcers, and stenosis. The total LS is the sum of the inflammation score of the proximal, middle, distal SB, and stricture. A score of <135 is compatible with normal or clinically insignificant mucosal changes, 135-790 defines mild mucosal and >790 defines moderate–severe mucosal disease as suggested.^19^

### Treatment and Follow-up

Adalimumab monotherapy was administered subcutaneously at baseline (week 0) at a dose of 160 mg, followed by 80 mg at week 2, and thereafter 40 mg every 2 weeks until 24 weeks of the visit. Therapeutic drug monitoring (TDM) and dosage adjustment were allowed during the maintenance therapy by the treating physician.

The patients were followed-up at weeks 0, 12, and 24 weeks and were assessed for clinical activity using the Harvey–Bradshaw index (HBI) for CD, while complete blood cell count, serum albumin, C-reactive protein (CRP), and fecal calprotectin (FC), and VCE were obtained at baseline and at week 24 of adalimumab treatment.

### Study Outcomes

The co-primary outcomes of the study were the proportions of patients with EH and/or endoscopic response (ER) induced by adalimumab at week 24. The EH was defined as an LS <350, and the completed EH was defined as an LS <135 on follow-up VCE. The ER was defined as a >50% decrease in total LS from baseline.^20^ Nonresponse was defined as a <50% decrease of total LS, need for corticosteroids due to flare of disease, or required surgery during the adalimumab treatment.

The primary and secondary outcomes were analyzed in the intention-to-treat (ITT) and the per-protocol (PP) populations. The patients who had baseline evaluation and received at least 1 dose of adalimumab therapy but had discontinued adalimumab treatment before completing the study, lost for follow-up, or did not have the second VCE were defined as nonresponders (not achieving the primary endpoint) in the ITT analysis. The secondary outcomes included clinical remission (HBI <4), biomarker remission including FC (<250 μg/g), and serum CRP (<5 mg/L).

### Statistical Analysis

Baseline demographic characteristics and categorical variables were presented as frequencies with percentages and compared using χ^2^ square or Fisher’s exact tests. Continuous variables were expressed as the median and interquartile range (IQR) and were compared using the *t*-test with separate variance estimates. Follow-up was calculated based on the date of the first adalimumab treatment. We performed univariable and multivariable logistic regression to estimate factors associated with EH. All statistical analyses were performed using SPSS software package version 20.0 (IBM Corp.; Armonk, NY, USA). *P *<.05 was considered statistically significant.

### Ethical Consideration

This study was approved by the Research Ethics Board (REB) of McGill University, under protocol REB No. 2011-3158 and No. 2014-1834. The written informed consent was obtained from all subjects and/or their legal guardians. Individual patient-level data were de-identified to maintain confidentiality in all steps of study analysis. This study was conducted in compliance with regulations stated in the 1975 Declaration of Helsinki.

## RESULTS

A total of 59 patients with known CD were screened with VCE for eligibility between November 2011 and June 2020. Of those, 22 patients had normal or mild SB mucosal inflammation (baseline LS <790). In the remaining 37 eligible CD patients with moderate–severe small intestinal mucosal inflammation, 20 patients were excluded due to refusal to escalate to biologics therapy (n = 6) or opted for biologic treatment other than adalimumab (n = 13) or failed patency VCE (n = 1).

Seventeen patients were enrolled in adalimumab treatment and included in the ITT analysis. Of those, 15 patients who had completed 24 weeks of adalimumab treatment and had a follow-up VCE were included in the PP analysis.

**Figure 1.**
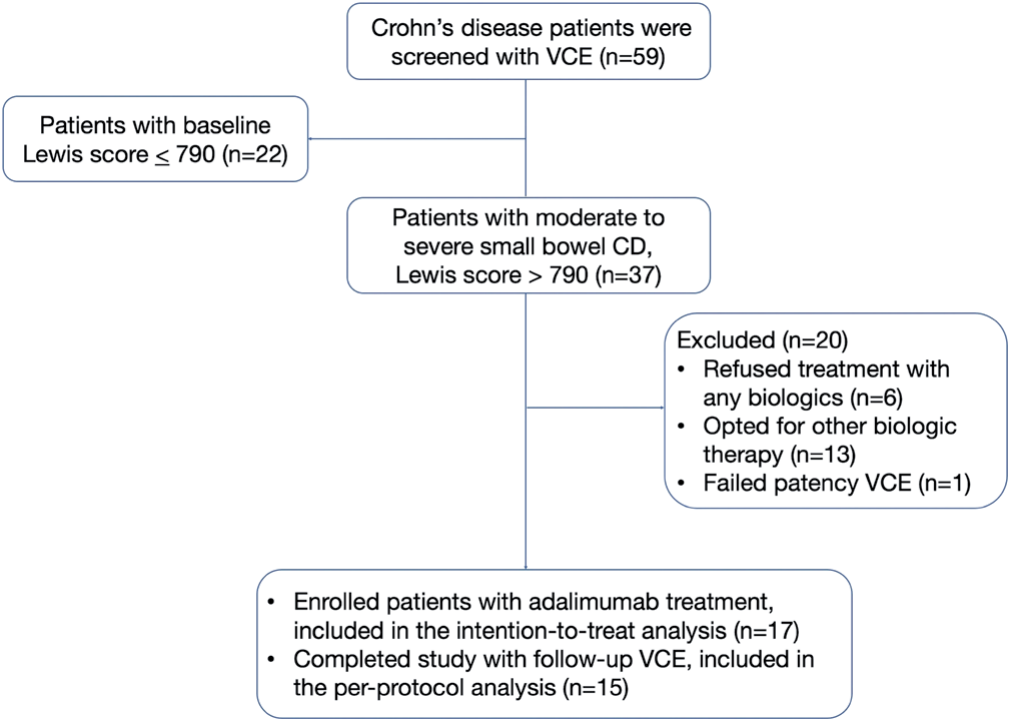
Study flowchart of SIMCHA study. SIMCHA, Adalimumab in Small Intestinal Crohn’s Disease.

The study flow chart is shown in Figure 1.

### Baseline Characteristics

Baseline characteristics of patients are described in Table 1. The median age at inclusion was 43.3 years (28.8-53.5), and the median duration of diagnosed CD was 7.5 months (2.8-51.0). The most frequent proximal SB CD involvement on VCE was the proximal–mid ileum (88.2%) and jejunum (76.5%). In addition to proximal SB CD, patients had coexisting ileocolonic (L3, 52.9%), terminal ileal (L1, 41.2%), or colonic (L2, 5.9%) disease.

All patients with coexisting colonic involvement had inactive endoscopic inflammation on conventional ileocolonoscopy at the time of enrollment (simple endoscopic score for CD, SES-CD <3). None of the patients had perianal disease nor previous intestinal resection. All patients had no other concomitant therapies for CD, including systemic corticosteroids and immunomodulators at enrollment. In addition, the TDM of adalimumab therapy was measured in 7 patients. One patient had a low through level and had dose optimization.

At baseline, the mean total LS on VCE was 1912 (IQR 1350-2080). The median HBI score was 6 (IQR 5-7). The biological markers: the median FC was 176 μg/g (IQR 132-431), and CRP was 7.4 mg/L (IQR 2.6-14.7).

### Primary Outcomes

In the ITT analysis, the EH was achieved in 8 of 17 patients (47.1%, *P* = .001 compared to baseline), and 4 of those with EH had completed EH (LS <135, a median LS of 112). Endoscopic response was found in additional 5 patients (29.4%). However, 4 of 17 patients (23.5%) have not achieved the primary endpoint. Two patients had no ER on the follow-up VCE. Two patients who failed adalimumab treatment before the end of follow-up and did not undergo the second VCE were defined as nonresponders, including 1 patient who had a loss of response and was switched to a different biological therapy and another patient who suffered a flare and required corticosteroids and surgery (Table 2).

Fifteen patients who had completed 24 weeks of adalimumab treatment and underwent follow-up VCE were included in the PP analysis. The EH was achieved in 8 of 15 patients (53.3%, *P* = .002 compared to baseline), and the ER was observed in 5 of 15 patients (33.3%).

**Figure 2.**
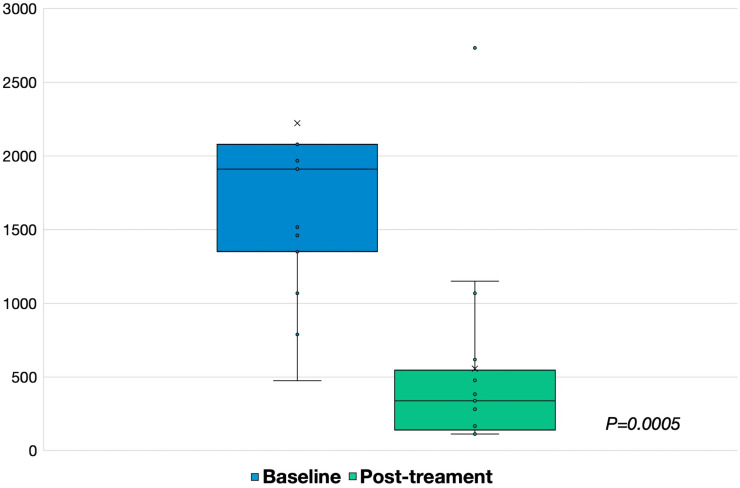
The Lewis score on VCE at baseline and after adalimumab treatment. VCE, video capsule endoscopy.

The median LS posttreatment with adalimumab at 24 weeks was significantly decreased compared to the LS at baseline (337 vs. 1912, *P* = .0005) in the PP analysis (Figure 2).

### Secondary Outcomes

#### Clinical Improvement:

**Figure 3.**
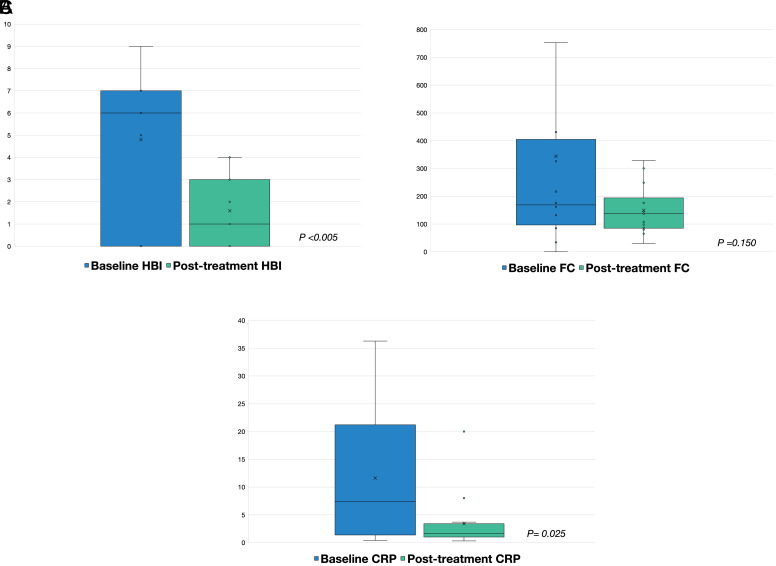
Secondary outcome on HBI, fecal calprotectin, and C-reactive protein at baseline and after 24 weeks of adalimumab treatment. (A) HBI, (B) fecal calprotectin, and (C) C-reactive protein. HBI, Harvey–Bradshaw Index.

The median HBI was significantly decreased after adalimumab treatment compared to the baseline HBI score [1 (range 0-4) vs. 6 (range 0-9), *P* < .005] (Figure 3). At baseline, 13 of 17 patients had active clinical activity (HBI >4). In the ITT analysis, 11 of 13 patients (84.6%) achieved clinical remission (HBI <4) after adalimumab treatment. (baseline: 13/17 vs. week 24: 2/17, *P* < .0001). In the PP analysis, 11 of 15 patients had clinical activity at baseline. All patients achieved clinical remission after 24 weeks of adalimumab treatment. (11/15 vs. 0/15, *P* < .0001).

#### Fecal Calprotectin:

The median posttreatment FC was not significantly decreased compared to the FC at baseline [138 (range 30-329) vs. 176 (range 34-1676) μg/g, *P* = .150]. Six patients had elevated FC at baseline (>250 μg/g). Of those, 4 patients achieved a normalized FC after treatment. In PP analysis, 4 of 5 patients (80%) with elevated FC at baseline achieved a normalized FC after 24 weeks of adalimumab treatment.

#### Serum C-Reactive Protein:

The median posttreatment serum CRP level was significantly decreased compared to the baseline [7.4 (range 0.4-20.5) vs. 1.6 (range 0.8-36.3) mg/L, *P* = .025]. Ten patients had elevated CRP (>5 mg/L) at baseline. Of those, 9 patients achieved normalized CRP levels after adalimumab treatment. However, 1 patient with a normal baseline CRP level had elevated levels posttreatment. In PP analysis, 7 of 8 patients (87.5%) with elevated CRP at baseline achieved CRP normalization after 24 weeks of adalimumab treatment.

In addition, we evaluated predictive factors associated with the SB EH, including age, gender, duration of CD, clinical, biomarkers activity, and serum albumin level. There was no significant factor associated with EH in patients treated with adalimumab, using univariate and multivariate analyses.

### Small Bowel Strictures

During the initial VCE, ulcerated SB strictures were found in 4 patients (23.5%) without capsule retention. Two out of 3 patients with follow-up VCE posttreatment were found to have persisting non-ulcerated strictures. However, 1 patient with SB strictures had a loss of response and needed another biological treatment before completing follow-up VCE.

## DISCUSSION

This prospective, open-label, single-arm study assessed the endoscopic MH and clinical efficacy of adalimumab treatment using VCE in biologic-naïve patients with moderate to severe, luminal, SB CD after 24 weeks. EH was achieved in 47% and ER in 29% of patients. We also showed the efficacy of adalimumab in inducing and maintaining clinical and biomarker remission.

Mucosal healing is an emerging concept in CD therapeutic monitoring. However, endoscopic MH of the SB in CD is difficult to achieve. In the VERSIFY study, patients treated with vedolizumab (VDZ) had lower healing rates in the terminal ileum (17%-25%) compared to the colon (40%-60%) in patients with moderate–severe endoscopic activity.^21^ It is important to note that 54% of patients included in the VERSIFY study were a prior anti-TNF failure, and those have a significantly worse clinical and ER to VDZ therapy. Similarly, in anti-TNF treated patients, the rates of SB EH were significantly lower compared to colonic healing rates as detected by balloon-assisted enteroscopy (36% vs. 79%, *P* < .001). In this latter study, 37% of patients had prior exposure to anti-TNFs, and 38% had concomitant azathioprine therapy.^9^ The results of this present study support that anti-TNF should be used preferentially in CD patients with proximal SB involvement. The clinical trials and real-world evidence have shown that anti-integrins and interleukin-12/23 are less effective in the second and higher line of biological therapy, especially in proximal CD.^22^

The rate of SB endoscopic MH in the present study was higher compared to the earlier studies, which used VCE for monitoring SB MH in patients with CD. Of note, all studies are single-arm observational studies. The study by Hall et al^15^ reported 28 CD patients with SB involvement treated with adalimumab/azathioprine. The reported EH rate was 42% at 52 weeks. Nonetheless, in this study, 33% of patients had only mild SB inflammation, and most of the patients (86%) had only ileal involvement, while very few patients had more proximal SB lesions. In another study, Niv et al^11^ used the LS to monitor SB mucosal response in 19 CD patients during different non-biologic treatments. While no patients achieved MH at 12 weeks of follow-up, the authors concluded that sequential VCE could be considered as a feasible and objective monitoring tool in SB CD. In a more recent observational study by Nakamura et al^23^ assessed MH in 29 patients with active mucosal CD (LS >135) on VCE, 79% of the patients had improved LS at 6 months after different additional treatments (5-ASA, immunomodulators, and anti-TNF).

Video capsule endoscopy is currently recommended for assessing SB inflammation but not as routine monitoring in the guidelines.^6,24^ Our study supports the use of VCE for objective therapeutic monitoring of SB healing and should be considered as a monitoring tool in CD patients where the SB is affected. In the present study, we used the LS, which has excellent, validated interobserver agreement in CD patients for SB mucosal lesions, with a sensitivity and positive predictive value of 82.6% and specificity and negative predictive value of 87.9%.^19,25^ Despite there is no standard optimal cutoff value of LS for EH, our study used cutoff <350 to determine EH since, in Ben-Horin et al.^20^ a LS of 350 or more was identified as the optimal cutoff value for predicting a disease flare within 24 months (HR 10.7, 95% CI 3.8-30.3; sensitivity 82%; specificity 77%; positive predictive value [PPV] 59%; negative predictive value [NPV] 92%).

The LS strongly correlates with capsule endoscopy Crohn’s disease activity index.^26^ In contrast, correlation with clinical activity, HBI score is poor (*r* = −0.15),^27^ and a low to moderate with CRP (*r* = 0.07 to 0.32) and FC (*r* = 0.044).^17,28^ In the present study, patients with endoscopically proven moderate to severe active SB inflammation on VCE, only 76%, 59%, and 35% of patients had active clinical symptoms, elevated CRP, and/or FC, respectively.

Although FC has high accuracy in assessing mucosal inflammation in colonic CD, a systematic review has shown that FC has more modest sensitivity (36%-77%) in detecting active inflammation in SB CD.^29^ C-reactive protein was reported to be more useful to discriminate inactive/mild from moderate–severe activity in SB CD.^30^ These findings further confirm that there is a partial disconnect between clinical symptoms, biomarkers, and mucosal inflammation in CD patients with SB inflammation, even in patients with moderate to severe luminal endoscopic activity. In addition, our data suggest that CRP rather than FC may better identify CD patients with active SB inflammation.

In the present study, ulcerated SB strictures were found in 4 patients at the initial VCE. After completing adalimumab treatment, these patients had no active inflammation but fibrotic lesions on VCE only. Of note, patients with known/symptomatic structuring disease and patients who failed patency capsules were excluded from the study. Previously, the efficacy of adalimumab in stricturing CD was investigated in a prospective observational cohort, including 97 CD patients with symptomatic SB stricture treated with adalimumab. Sixty-four percent of treated patients avoided additional interventions (need for corticosteroid, endoscopic dilation, or bowel resection) during a 24-week follow-up.^31^

The strength of our study was that the MH of extensive, moderate-to-severe, luminal, SB inflammatory CD were evaluated prospectively and objectively by repeated VCE, clinical, and biomarker assessment in biologic-naïve early diagnosed CD patients on adalimumab therapy, the first study of its kind. In addition, VCE assessment pre- and posttreatment was performed by a single investigator blinded to clinical results, earlier VCE results (if available), and patient characteristics to avoid/decrease intra-observer and inter-observer bias.

Nonetheless, there were limitations to this study. First, the number of patients included in the study was small. The number of patients screened was substantial, but the patients refused the biological therapy, took part in a clinical trial, or opted for another biological or molecule therapy. Finally, approximately one-third of the screened patients did not meet the preset VCE criteria for endoscopic activity. Therefore, the findings should be interpreted with caution. Second, there was no control group, and all patients received active treatment. Furthermore, patients with symptomatic stenosis, earlier surgeries, or failed patency capsules were excluded. Thus results can only be extrapolated to patients with luminal, noncomplicated proximal SB CD. Finally, follow-up was stopped at 24 weeks; consequently, long-term mucosal and clinical outcomes could not be assessed.

In conclusion, the SIMCHA study showed that proximal SB CD patients had significant improvement in SB mucosal inflammation during adalimumab treatment, with 47% of patients achieving EH and 29% ER at 24 weeks. Adalimumab also led to significant clinical remission and biomarker normalization. Finally, our data support the use of VCE in routine monitoring of SB MH as the optimal and objective treatment target in patients with proximal small bowel CD.

## Figures and Tables

**Table 1.** Baseline Characteristics of the Patients with Proximal Small Bowel Crohn’s Disease

Characteristics	Overall (n = 17)
Age (years), median (IQR)	43.3 (28.8-53.5)
Male gender, n (%)	9 (52.9%)
Disease duration at enrollment (months), median (IQR)	7.5 (2.8-51.0)
Active smoking at diagnosis, n (%)	1 (5.9%)
Concomitant therapy for CD, n (%)	0
Previous exposure to biologics	0
Crohn’s disease phenotype, n (%)	
Age at diagnosis (A1, A2, and A3)	2 (11.8%), 7 (41.2%), and 8 (47.0%)
Location of upper GI tract involvement, n (%)	
Proximal–mid ileum	15 (88.2%)
Jejunum	13 (76.5%)
Duodenum	2 (11.8%)
Gastric	1 (6.7%)
Coexisting terminal ileal (L1), colonic (L2), and ileocolonic (L3) involvement	7 (41.2%), 1 (5.9%), and 9 (52.9%)
Strictures, n (%)	4 (23.5%)
Previous intestinal resection	0
Perianal involvement/fistula	0
Extra-intestinal manifestation	1 (6.7%)
Comorbidities and other autoimmune-related diseases, n (%)	
Ankylosing spondylitis	8 (53.3%)
Diabetes mellitus (DM) type 2	3 (20.0%)
Psoriasis/psoriatic arthritis	2 (13.3%)
Rheumatoid arthritis	1 (6.7%)
Hashimoto thyroiditis	1 (6.7%)
Chronic renal disease	1 (6.7%)
Crohn’s disease activity at baseline, median (IQR)	
Clinical score (HBI)	6 (5-7)
Hemoglobin, g/L	138.0 (126.3-151.5)
Albumin, g/L	39 (35-42)
CRP, mg/L^a^	7.4 (2.6-14.7)
Fecal calprotectin, μg/g	176 (132-431)
Lewis score on VCE at baseline	
First quartile	730 (224-1518)
Second quartile	1350 (871-1968)
Third quartile	1068 (575-1604)
Total score	1912 (1350-2080)

CD classification: A1, age (years old) at diagnosis <17; A2, 17-40 years; A3, >40 years; B1, non-stricturing, non-penetrating; B2, structuring; B3, penetrating; L1, terminal ileum; L2, colon; L3, ileocolonic; L4, upper gastrointestinal CD.

CD, Crohn’s disease; CRP, C-reactive protein; HBI, Harvey–Bradshaw Index; IQR, interquartile range; VCE, video capsule enteroscopy.

^a^Normal value for CRP was 0-5 mg/L.

**Table 2.** Primary Outcomes in EH or ER After Adalimumab Treatment as the ITT Analysis

Primary Outcomes	Number of Patients (%)
Endoscopic healing (Lewis score <350)	8 (47.1%)
Endoscopic response (>50% reduction in Lewis score)	5 (29.4%)
Non-responders	4 (23.5%)

EH, endoscopic healing; ER, endoscopic response; ITT, intention to treat.
